# Improving Communication between Physicians and Their Patients through Mindfulness and Compassion-Based Strategies: A Narrative Review

**DOI:** 10.3390/jcm6030033

**Published:** 2017-03-17

**Authors:** Alberto Amutio-Kareaga, Javier García-Campayo, Luis Carlos Delgado, Daniel Hermosilla, Cristina Martínez-Taboada

**Affiliations:** 1 Department of Social Psychology and Methodology of the Behavioral Sciences, University of the Basque Country (UPV/EHU), Leioa 48940, Spain; 2 Department of Psychiatry, Miguel Servet University Hospital, University of Zaragoza, Zaragoza 50009, Spain; jgarcamp@gmail.com; 3 Department of Evolutionary and Educational Psychology, University of Granada, Melilla 52071, Spain; lcpastor@ugr.es; 4 Department of Social Psychology and Methodology of the Behavioral Sciences, University of the Basque Country (UPV/EHU), Donostia-San Sebastian 20018, Spain; daniel.hermosilla@ehu.eus (D.H.); cristinamtk@gmail.com (C.M.-T.)

**Keywords:** physician-patient relations, psychosocial support, mindfulness and compassion, communication, well-being

## Abstract

Communication between physicians and patients is a key pillar of psychosocial support for enhancing the healing process of patients and for increasing their well-being and quality of life. Physicians and other health professionals might benefit from interventions that increase their self-care, awareness, compassion, and other-focused concern, and reduce the chances of distress and burnout. There is substantial evidence for the contribution of different management strategies to achieve these aims. The goal of this article is to review the potential effect of mindfulness and compassion-based strategies for the improvement of physician-patient interactions. The acquisition of the necessary skills by physicians requires continuous education. Future research will be useful for identifying more evidence on the cost-effectiveness of this type of intervention.

## 1. Introduction

### 1.1. The Risk of Burnout

Health care professionals (HCP) are particularly vulnerable to stress and empathy/compassion fatigue, due to an emotionally exhausting environment and exposure to the suffering of patients [1,2]. There are numerous sources of stress for clinicians. Some are external (e.g., heavy patient load, time pressures, having to make important medical decisions, interpersonal staff conflict, lack of autonomy in the work environment, record-keeping requirements, financial concerns, and difficulty with balancing personal and professional lifes [3,4]). Other stressors are internal, such as personality characteristics (e.g., perfectionism), harsh self-judgment, and poor emotional regulation [5]. In this way, effective emotion regulation is essential for physicians exposed to the suffering of others, because it dampens counterproductive feelings of alarm and fear, and frees up the processing capacity to be of assistance to the other person [6].

Previous research has widely described the relationship between stress, burnout, the absence of self-compassion, and compassion fatigue (CF) among health professionals [7,8,9,10]. Studies from the United States and different countries in Europe report a prevalence of burnout from 27% up to 45% among practicing physicians, depending on the specialty, and especially note the prevalence of emotional exhaustion [3,4,11,12]. Depending on the specialty, between 20% and 70% of physicians suffer from CF, a state of emotional exhaustion and diminished empathy brought on by the unceasing demands of patient care [13]. Burnout and CF among caregivers, in turn, has been associated with a less effective delivery of care and decreased patient satisfaction [1,14].

Communication with patients (and their families) is a key issue in healthcare. This is especially true for some diagnoses, for example, cancer, HIV, or diabetes. In these cases, some of the physician’s communicative behaviours are of paramount importance, such as: expressing empathy, allowing for a sufficient period of time so that patients can absorb the diagnosis, providing information in a way that is fully understood by the patient, and engaging the patient in treatment decision-making [15,16,17].

A well-developed patient-physician relationship has been shown to improve patient health outcomes and compliance. Being empathic is one of the many factors contributing to the enhancement of the patient-physician relationship [18]. In the clinical setting, empathy includes the ability to listen to a patient, understand their perspective, sympathize with their experience, and express understanding, respect, and support. However, empathy declines dramatically as medical students progress through medical school, and this change coincides with students’ and medical residents’ reports of high rates of burnout and psychological distress [19,20].

A systematic review concluded that physician empathy is associated with beneficial outcomes, based on patient-report measures in cancer care [21]. Consequently, the reported shortage of empathy and the decline in empathy during medical training [22], only amplifies the importance of finding reliable interventions for physicians and physicians-in-training. Indeed, heightened empathy among medical practitioners could not only lead to a more ethical healthcare system, but could also result in the enhanced health and well-being of both patients and practitioners [23].

An increasing number of investigations have found positive relationships between clinical-patient communication, treatment compliance, and a variety of health outcomes, including a better emotional well-being, lower stress and burnout symptoms, lower blood pressure, and a better quality of life (for both doctors and patients) [4,24,25]. However, poor communication between health care professionals and their patients is a key issue in the increasing number of complaints against health care professionals worldwide. Yet, according to a systematic review by Griffin et al. [26], only 44% of interventions designed to enhance the interaction between patients and physicians and stimulate patient participation had significant positive effects on the outcomes. Hence, the general idea that merely activating patients to become more participative will lead to better outcomes does require further clarification. A more fruitful approach might be to match patients’ preferred levels of participation with their actual participation levels. In Schinkel et al.’s [27], study, the match between preferred and perceived patient participation was a strong predictor of communication outcomes, whereas mere doctor-patient concordance was not. Therefore, doctors need to become more aware of both cultural and individual differences in patients’ participation preferences and need to adjust their communicative behavior to these differences. This is where HCP’s empathy and compassion levels play a fundamental role. Taking this situation into account, interventions to improve patient-professional communication have included enhancing practitioners’ empathy as one of the core components [28].

### 1.2. Mindfulness and Compassion: Conceptualizations

One of the mind-body interventions available for work stress and other stress-related problems is mindfulness meditation. Mindfulness can be described as non-judgmental attention to experiences of the present moment, including emotions, cognitions, and bodily sensations, as well as external stimuli [29,30]. It is both a practice and a way of being in the world, in which the individuals maintain attitudes such as openness, curiosity, patience, and acceptance, while focusing their attention on a situation as it unfolds. Thus, mindfulness is congruent with the overarching goal in medical practice to cure disease when possible and meet suffering in a compassionate manner [31]. In this way, mindfulness can be seen as a set of skills that facilitates the healing aspects of the clinician-patient encounter [32].

Bishop et al. [33] proposed a two-component model of mindfulness: self-regulation of attention and the acceptance of one’s own experiences (non-reactive awareness). Overall, mindfulness techniques have proved to be successful in the treatment of a wide array of physical, psychosomatic, and psychosocial dysfunctions [34,35,36,37], and there is a growing body of research demonstrating the effectiveness of mindfulness and compassion-based meditation practices for reducing the physical and psychological effects of stress among health care workers [38,39].

Mindfulness interventions designed for clinical settings were originally developed in the United States, and currently there is a widespread interest in them in many countries. According to a recent study carried out by Barnes et al. [40] in US medical schools, 31% of these schools offer mindfulness opportunities within their curriculums. The most commonly offered program was Mindfulness Meditation, and *Mindfulness-Based Stress Reduction* (MBSR) [29]. However, the United Kingdom is apparently the most developed country in terms of the formal implementation of Mindfulness-based interventions (MBIs), specially Mindfulness-Based Cognitive Therapy (MBCT) in an integrated national healthcare system, involving institutional support in terms of funding and training of human resources [41].

MBSR [29] has been successfully applied to healthcare professionals to reduce stress-related symptoms, including burnout [42,43], and to increase self-compassion, resilience, and well-being [44,45]. A modified and extended MBSR program for primary physicians offered by Krasner et al. [46], resulted in self-reported improvements in mindfulness, burnout, empathy and responsiveness to psychosocial aspects of patients’ problems, mood disturbance, emotional stability, conscientiousness, and engagement at work. Subsequently, various types of psycho-educational interventions based on MBSR for treating occupational stress in health professionals in different countries have been undertaken, and there are different studies around the world substantiating this conclusion, including: Spain [7,47,48], the USA [12,44], France [49], Canada [50], Colombia [51], and Chile [34]. All of these studies point to the benefits of training physicians to be able to better handle stress and their emotions, and also to communicate with more awareness when dealing with patients.

Mindfulness-based interventions (MBIs) have also been successfully applied to other professionals, including teachers and students [52], and even the general population [53,54]. Additionally, briefer mindfulness interventions are also taking place. In this sense, Brito [34] and Hevezi [55] obtained significant increments in compassion in a sample of 17 nurses using a meditation intervention lasting four weeks. These results are important given the fact that health professionals frequently report difficulties in finding enough time to learn relaxation and meditation techniques. Further research is needed to truly verify the efficacy of shorter interventions.

Historically, most psychosocial intervention research has focused on the alleviation of negative emotional states. However, there is growing interest in cultivating positive emotional states and qualities. Recently, compassion-based therapies are emerging. Compassion requires an awareness of the other’s suffering and a toleration of the distress associated with the witnessing of suffering. It entails the feeling of affect or proximity towards other human beings, and the wish that they remain free from suffering [39]. Compassion contributes a crucial aspect that can make empathy truly therapeutic: a desire to relieve suffering and a preparation for action [56,57]. Enacted compassion appears to be associated with a sense of reward, meaning, and purpose [58]. Physician compassion is a key element in good doctor-patient relationships; compassion is nourishing for the healer, as well as the patient [59]. Having compassion for others entails self-compassion [10,60]. Self-compassion may be an important skill to teach to caregivers and health professionals, as it can be seen as a first step in cultivating compassion care for patients, and this is where mindfulness comes into play.

Mindfulness and compassion are closely interrelated, as mindful attention includes a compassionate quality within the attending, a sense of an openhearted friendly presence, and interest [29,30]. Improved self-care can decrease CF and burnout, while increasing compassion satisfaction; that is, the positive feeling associated with knowing that the professional has, in some way, helped another [30]. Accordingly, a review by Sorenson et al. [61] concluded that self-care was the most significant preventive measure that health care providers could take to protect themselves from developing CF and burnout. Both mindfulness and (self) compassion independently accounted for significant components of the effect of meditation experience on psychological well-being [30]. Working in a hospital environment that embraces compassion-based values yields higher employee well-being and maintains organizational commitment [62].

Continuing education about self-management techniques, including stress management and (self)-compassion, is necessary. Yet, many mental health and medical providers may not be familiar with mindfulness-based approaches to physical, social, or psychological health, despite the fact that these skills can be cultivated by anyone. Besides, HCPs in general, neglect the emotional demands derived from their working conditions (traumatic tasks, working with people and their emotions, continuous changes, etc.), and do not receive adequate organizational support, which has important consequences for their own well-being and health that end up being reflected in an impoverishment of the quality of care.

Currently, there is a paucity of studies on the effectiveness of MBIs for improving interactions between physicians and patients. The aim of this narrative review is to highlight the potential effect of mindfulness and compassion-based strategies to enhance the physician-patient relationship and to indicate future venues for research, teaching, and practice.

## 2. Methods

### 2.1. Selection Criteria

Original research and review articles on the topic of mindfulness and compassion strategies in relation to physician-patient interactions were included in this narrative review. A yearly range was not established given that the topic is relatively new and because we wanted to find what was in the literature as a whole. Papers examining different samples of HCPs were included. The articles reviewed were mainly in English.

### 2.2. Search Strategy

Different databases: PubMed, MedLine, Google scholar, Scopus, ScienceDirect, PsychInfo, and Social Sciences Abstracts were used to search for articles in medicine and psychology journals, guided by search terms related to the topic. Different key words in Spanish and English were entered, including: Mindfulness-based interventions, Compassion-based interventions, Physician-patient interactions, Physicians’ burnout, Patient-centered care, Patient’s well-being, and Psychosocial support. The selected literature was then systemically reviewed and synthesized for this narrative review.

### 2.3. Procedure

The authors independently performed research for articles using the different descriptors and key words, and the title and abstract. The full texts of these studies were retrieved and assessed for eligibility using the inclusion criteria outlined above, yielding 75 eligible studies which met the established criteria. Additionally, we reviewed the references of the selected articles and included 40 more studies using this method. Members of the research team (AAK, LCD, JGC, DH, and CMT) independently examined the full texts of studies that passed the title and abstract review and met the inclusion criteria. A total of 115 articles were examined. Each article was assessed for a variety of metrics, including source population, sample size, type of intervention, duration of intervention, assessment strategy, type of outcome measure, and outcome assessment time frame. Finally, a total of 20 articles met the full criteria of including quantitative and qualitative data, or review studies on the effectiveness of mindfulness and compassion-based techniques in physician-patient communication. Data were extracted and finalized through discussion within the research team.

## 3. Results

The results obtained following our search strategy are included in Table 1.

Concerning the efficacy of mindfulness and compassion-based interventions for practicing physicians, MBIs have been correlated with decreased burnout and improved empathy rates [7,24,46,63,75]. MBIs represent a rapidly growing area of medical research and practice that can potentially benefit both physicians and patients [40,76]. In a study conducted by Beach et al. [42] with a sample of 45 clinicians and 437 HIV-infected patients, mindfulness among health care clinicians was associated with more patient-centered communication, providing the first evidence of a link between clinician mindfulness and quality of care patients received, as measured by patient-centeredness and patient satisfaction. Specifically, mindfulness among clinicians was associated with an increase in rapport building and communication of psychosocial issues between patient and clinician. Thus, mindful practitioners can learn to listen attentively, develop a deeper appreciation of the patient’s experience, and respond with empathy and compassion. Patients, for their part, then feel empowered to make their voice heard in areas that matter to them. This is one of the few studies in this area which uses the reports of its own patients.

Dean & Street [67] introduce a three-stage model of patient-centered communication for addressing emotional distress in cancer patients: Recognition, Exploration, and Therapeutic Action. Recognizing the patient’s emotional distress requires cognitive strategies (e.g., mindfulness) and communicative strategies (e.g., active listening). Exploration entails a mindful non-judgemental attitude and empathy. Here, mindfulness can help the clinician to be self-aware and attentive to the patient, reacting with curiosity, openness, and equanimity. Therapeutic action includes making medical decisions and taking steps regarding self-care. Communication training for clinicians needs to include all stages of this model. Some of the key contents of these sessions for clinicians could focus on mindfulness, active listening techniques, and empathy. This model fills an important gap in the literature.

A unique, individualized short-term psychotherapy is Dignity Therapy [77]. The therapeutic process begins with a framework of questions that are based on an empirical model of dignity in the terminally ill patient. These conversations, guided by a trained therapist, are flexible, in order to accommodate the patients’ needs and choices about what they specifically wish to address. In a study (*n* = 441) conducted by these authors, patients reported that Dignity therapy was significantly more helpful for increasing their quality of life, more helpful for their families, and was better at improving spiritual well-being than standard palliative care and client-centered care. In conclusion, its benefits in terms of self-reported end-of-life experiences (e.g., better psychosocial care) support its clinical application for patients nearing death.

Some authors (e.g., [64,78]) describe how mindfulness can enhance the therapeutic relationship by cultivating unconditional positive regard and empathetic understanding towards the patient. An example is the Mindfulness-Based Medical Practice program [68], where regular meditation practice enabled clinicians to be more open-minded and compassionate toward themselves and their patients. In a focused group mindfulness study, healthcare professionals reported that the course increased their ability to be non-judgmentally aware and accepting of their thoughts, sensations, and emotions in clinical encounters, resulting in better listening skills and more empathy for their patients.

Another example is Mindful Communication [46,63]. The program significantly improved the indicators of patient-centered care (e.g., empathy, psychosocial orientation), while also enhancing the physician’s well-being (e.g., decreased burnout, improved mood). These changes were mediated by changes in the physician’s mindfulness levels. Amutio et al. [7,24] obtained decreases in burnout in a controlled study with 42 physicians after a one-year intervention. The intervention followed the MBSR program [29] and was based on the psycho-educational model of Krasner et al. [46]. Additionally, a positive and significant relationship between the benefits reported by the practice of mindfulness and the improvements of physicians’ understanding and communication with patients (*r* = 0.490; *p* = 0.033), was obtained. Also, physicians reported an improvement in interpersonal communication, which was associated with a better management of stressful situations. This is one of the few longitudinal studies in the area.

Halifax [79] has developed a model for developing compassion among nurses. The intervention is called GRACE and has five stages: Gathering attention (get grounded and stay focus); Recalling intention (affirming meaning and purpose: preserve the well-being and integrity of the patient); Attunement (first to oneself, and then to the patient or family member); considering what will serve the patient; and Enacting (engaging or applying compassion in service to others).

Additionally, research suggests that mindfulness interventions, particularly those with an added loving kindness component, have the potential to reduce CF and distress, and increase self-compassion, feelings of empathy and social connectedness, and emotional regulation among health care workers [55,80]. Some pilot studies have showed the beneficial effects of the practice of compassion, especially for health problems related to excessive self-criticism and guilt that can be found in some subtypes of burnout, and dysfunctional interpersonal relationships (e.g., social phobia). An extensive review [74] has shown that Loving-kindness Meditation—LKM and Compassion Meditation (CM) produce significant clinical improvements in different areas, including: increments in positive affect and decreases in negative affect, increases in positive thinking, more adaptive interpersonal relationships, and the development of empathy between professionals and patients. This result is in line with other studies in the area [64,81].

In a recent revision by Lamothe et al. [69], they identify a number of MBSR-based studies reporting an increase in emotional competencies of major importance for high quality care, including emotional acceptance, the identification of one’s own emotions, empathy, and self-compassion. However, there is a paucity of results on these variables. In general, these authors conclude that, although it is possible to attribute the effect of MBSR on healthcare providers to mindfulness per se, there are not any studies examining the mediation effects of mindfulness on the studied outcomes. Nevertheless, the idea that physical and mental outcomes are favourably influenced by mindfulness practice is strong.

## 4. Discussion

To our knowledge, this is the first review focused on the efficacy of mindfulness and compassion-based strategies for improving communication between health care professionals and their patients. Previous interventions and reviews have been more focused on reducing distress in health professionals and improving well-being. Stress reduction programs focus on cognitive-behavioral techniques, relaxation, and mindfulness. They provide a combination of psycho-educational treatment combined with homework and follow-up sessions. Results of systematic reviews that evaluated stress management strategies (e.g., relaxation, mindfulness, exercise) among physicians reported that they are effective [2,37]. Moreover, group methods are both more cost-effective and more beneficial than individual counselling sessions [4].

The results of our review suggest that mindfulness and compassion-based strategies are effective for improving communication between physicians and their patients, enhancing physician’s empathy and quality of care. Being empathic is a crucial factor contributing to enhancing the patient–physician relationship. Empathy has been named as an essential learning objective by the American Association of Medical Colleges and it is believed to significantly influence satisfaction in patients, health care professionals, and with clinical outcomes [82]. Empathy may be considered an acquired skill that can be developed and refined. This ability to appreciate how similar circumstances may affect individuals differently is an essential trait for physicians, who must tailor treatments to achieve the personal goals of each patient. Empathy is also beneficial for the provider, as it corresponds to higher rates of job satisfaction and improved clinical practice [18]. Yet, while the importance of empathy as a core competency for physicians is well recognized, the way in which to promote the development of this skill is relatively unknown [19]. We propose that mindfulness-compassion training is a potential tool to enhance empathy towards patients. Mindfulness introduced into the curricula is a cost-effective way to provide students with the interpersonal skills which are essential for leading them towards positive practices during their studies and beyond, into their professional practice. Although patient-centered communication skills must still be learned, the practice of mindfulness may make it more likely that the clinician is able to employ this technique [42].

Despite our findings, this study has some limitations, mainly due to the fact that it is a narrative review. However, this is a first step. More original research on the efficacy of mindfulness and compassion-based interventions to improve communication between physicians and their patients is needed before more systematic or meta-analytical reviews can be undertaken. Another limitation is that most of the studies are based on subjective reports of health professionals, rather than on patient reports. Finally, sample sizes of the included studies were relatively small. Potentially limiting factors may also have been introduced by the eligibility criteria employed in the current narrative review. More specifically, English language studies were mostly included, which, given the popularity of Buddhist-derived meditation techniques in Eastern-language countries, may have resulted in the omission of relevant empirical evidence. Likewise, Doctoral dissertations or unpublished papers were not included in the review, meaning that further potentially relevant evidence may have been disregarded.

### Mediating Mechanisms

As mentioned above, there is a lack of investigations on mediation effects. However, many mediating mechanisms (psychological and neurobiological) for the efficacy of mindfulness and compassion-based interventions have been proposed. See Table 2.

A reasonable hypothesis would be that mindfulness-compassion meditation helps psychotherapists and clinicians self-regulate their emotions while they are attuned to their client’s suffering, reducing the burnout linked to empathetic distress or fatigue, and enhancing concern for others, altruism, and a more genuine empathetic communication [64,69,89]. Functional and structural MRI studies show consistent changes following mindfulness meditation training in core regions associated with the self-regulation of attention and emotion, including the anterior cingulate cortex, and the lateral and medial prefrontal cortex [76,87]. Thus, the physician’s ability to take the patient’s perspective has been found to be correlated with increased brain activation in the right ventrolateral and dorsolateral prefrontal cortices, and the rostral anterior cingulated cortex, regions associated with increased attention during the treatment of patients, expectancy for pain-relief, rewards-processing, and subjective value [90].

Some studies [91] seem to support differences in amygdala activation between mindfulness and compassion meditation. In this sense, compassion meditation has been found to be correlated with higher amygdala activation and a higher heart rate, as compared to mindful-attention, suggesting that the compassion meditative state was one of higher arousal. These findings may support the idea of higher other-focused concern in compassion meditation.

Mindfulness is often considered a precursor to the development of compassion. Conceptually, the cultivation of compassion is thought to rely on stabilizing the mind via mindful awareness practices. However, compassion training may also enhance mindfulness [92,93]. Furthermore, increases in self-compassion have been found to be a mediator in stress reduction, attenuating the link between burnout and distress [94]. Thus, self-compassion appears to be a major therapeutic factor in mindfulness-based interventions [95,96].

Today, there are still contradictory findings across different studies and it remains unclear what elements of communication are associated with specific outcomes [25]. One of the most important challenges in this area consists of determining the mediating mechanisms or communication pathways which improve health [97]. For example, effective clinician-patient communication may produce immediate outcomes (e.g., better patient understanding of treatment options, or physician understanding of patient preferences), which in turn contribute to more intermediate outcomes (e.g., better adherence, self-care skills), thus improving the outcome of interest (e.g., psychological well-being, reduced anxiety, and pain). However, communication research suffers from a serious lack of integration across different research programs, both in terms of theory and measurement. So, it is important that researchers identify the specific communication processes that need to be activated in order to achieve the desired outcomes. In this way, future interventions need to target the communication variables or factors that could activate the pathway to improved health and well-being. Besides, physicians’ awareness and sensitivity to minority patients and cultural differences will be crucial in this process [27].

Despite the complexity of the communication process in medical encounters, patient-centered care improves patients’ well-being. It can do so directly, through reducing anxiety and depression, and also indirectly, by building trust and social support. These outcomes, in turn, increase patients’ ability to cope with adversity, while also promoting access, understanding, and adherence. They also promote patient self-efficacy, enabling patients to navigate the health care system more effectively [25].

## 5. Practical Issues

Some steps for improving physician-patient communication are presented (Table 3).

Mindfulness-Compassion practices can be of help in this process, given the fact that active listening implies that health professionals listen with an open mind, remain focused on the client’s message, and resist judgment.

Additional strategies involving health professional’s work at the personal level include:

- Being aware of experienced emotions (e.g., frustration, anger, hopelessness).
- Having a non-judgmental attitude towards the patient.
- Self-care (e.g., maintaining a balance between one’s personal and professional life, relaxation-mindfulness).

See Table 4 for an example of a brief mindfulness technique (three-minute breathing space) that can be practiced first by the therapist, and then with the patient or client during the session, to improve communication and treatment effectiveness.

## 6. Future Needs

Neuroscientific studies are needed to identify the role of workplace factors in empathy decline [102]. Additionally, well-designed, rigorous, and large-scale RCTs are necessary to decisively provide estimates of the efficacy of mindfulness and compassion-based interventions for enhancing empathy, compassionate care, and improving physician-patient communication. Future studies will need to incorporate some methodological improvements [40,69,103], including:

- Sufficient sample size.
- Randomized control trials.
- The use of clinical samples, or patients needing the intervention.
- Using not only physicians’ reports, but also patients’ reports and clinical outcomes.
- The inclusion of emotional competencies as outcome variables
- Measuring the effectiveness of shortened versions of mindfulness programs.
- Research on the nature of compassion and its implications in clinical practice with patients
- Verifying mediating mechanisms and communication pathways.
- The use of quantitative and qualitative methodologies.
- Evaluating the extent to which medical students and physicians utilize the MBIs available at their institutions, or the extent to which mindfulness courses are required versus elective ones
- Longitudinal studies and active follow-ups.

## 7. Conclusions

Modern Western healthcare, with its emphasis on technology, often results in the patient being ignored as a person. Over the last ten years, the volume of patient complaints has more than doubled. Communication- and information-related issues were the second most commonly complained issue in relation to both public and private hospitals, across all medical specialties [28]. Mindfulness practice could reverse this process, since it assists in the development of self-awareness and emotional regulation skills, which enables students and health professionals to be more open to perspective taking and, consequently, allows them to show more empathetic concern. Mindfulness may enhance patient-centeredness in clinical encounters by improving both the quantity and the quality of the attention which clinicians give to their patients. According to Beach et al. [42], greater patient-centeredness and improved patient experiences with mindful clinicians could ultimately enhance retention in care and clinical outcomes, as for example, in the setting of HIV care, where positive patient-clinician relationships have been linked to higher quality, greater medication adherence, and better outcomes. Similar results were obtained by Fuertes et al. [104], with a sample of 101 adult outpatients from a rheumatology clinic. Results demonstrated that the physician-patient working alliance predicted outcome expectations, patient satisfaction, and adherence. These benefits may extend to all patients, especially to those from marginalized populations and ethnic minorities, for whom trust and respect are central issues.

Person-centered communication practices improve both clinical outcomes and patient safety in healthcare settings. Patients, especially those with chronic illness, need to be empowered to take responsibility for their health, be assertive with their caregivers, and be conscious of how they influence the doctor-patient relationships. Zoppi and Epstein [105] referred to this as “intersubjectivity”, which includes the creation of shared meanings: goals, beliefs, and intentions concerning their work together.

In addition to MBIs, other plausible alternatives are balint sessions. Balint sessions are group sessions that train doctors on how to apply a patient-centered approach, with a special focus on doctor-patient relationships [4]. However, interventional studies evaluating the effectiveness of Balint groups are scarce. Other types of interventions include communication skills courses, which are basic for health care professionals. Yet, traditional medical programs do not introduce communication skills training until the last stages of medical training [22]. Relaxation and stress management techniques, group analytical therapy, and other psychotherapeutic techniques can also be effective options. However, the obtained results are not conclusive and more studies are needed [106].

Organizational culture may be a barrier to person-centered communication and patients’ engagement. Hence, organization-wide approaches are needed to implement person-centered care interventions, such as mindfulness [107]. Additionally, health care delivery systems must implement systematic change at the practice level to create an environment that supports mindfulness practice. Thus, managers should aim to create a professional environment that promotes teamwork and positive working relationships [61]. Mindful efforts to improve the healthcare culture and develop personal support systems can help physicians become more resilient and provide higher quality patient care. Managers and policy makers should focus on improving the functioning of relevant human resources management systems in health care. Institutional support systems are fundamental [108].

Along with mastering the biomedical knowledge required by the profession, a paradigm-shift to a culture where professionals are willing to attend to work on self-awareness and mindfulness is necessary [109]. This includes: providing training to employees to produce higher levels of knowledge and skills relevant to patient care; encouraging clear communication among clinicians, staff, patients, and families; and reducing isolation [63]. Given that the acceptability of MBIs among health professionals is high [7,110], continuing education for health professionals would also be highly feasible A combination of both personal and organizational interventions will have longer lasting positive effects [11].

Enhancing the focus on developing self-compassion using MBSR and other mindfulness-compassion interventions for health care workers holds promise for reducing perceived stress and increasing the effectiveness of clinical care [1,65,111]. Self-compassion may protect against burnout and empathy/compassion fatigue. Additionally, mindfulness practice can improve physician-patient therapeutic relationships, specifically enabling practitioners to listen more attentively, be aware of their own mental processes, recognize bias and judgments in thinking, and develop more effective communication practices [80]. By using mindfulness techniques, practitioners develop increased compassion, more effective physician-patient communication, and improved patient outcomes.

There is ample evidence for the effectiveness of these programs in preventing burnout, although more randomized controlled trials with bigger samples and follow-ups are needed. In the current climate of managed care and increased emphasis on efficiency and cost containment, the fact that conveying compassion requires so little time is important. Fogarty et al.’s [17] study with 123 healthy female breast cancer survivors found that being perceived as compassionate, and thereby influencing anxiety levels and perceptions of physician attributes, can take less than 40 s. Teaching interventions can effectively change physicians’ behaviours and increase their emotional skills, without increasing the length of the visit and resulting in a sustained impact on patients’ emotional distress.

Physicians’ positive beliefs about psychosocial aspects of patient care are associated with better communication with patients during routine office visits. Patients of physicians with more positive attitudes are more involved as partners in their care. These findings have implications for medical educators, teachers, and practicing physicians [99,112]. However, it is possible that MBIs may be underutilized, due to a lack of knowledge among health professionals and students of health professions. The results of some studies demonstrate that a knowledge of mindfulness is associated with an increased positivity towards it, and an expressed willingness to administer or recommend it [48,113].

Mindfulness programs directly focus on giving providers the tools to combat the many stressors they face on a daily basis and to promote resilience and well-being. Evidence is mounting that mindful Medical Practice enables clinicians to offer Whole Person Care [32]. Compassionate care enhancement represents a key factor in healthcare reform [114]. By drawing attention to the effects of mindfulness and compassion-based interventions in enhancing physician-patient communication, we hope to stimulate the implementation of these kinds of programs in medical schools and universities, and to encourage new research.

We would like to end this review with a quote (Beach et al. [42], p. 427):

Mindfulness may be an important pathway to a more humanistic, effective, and satisfying practice of medicine. The highly reciprocal influence of patients and clinicians on one another is in itself a powerful and positive medical tool—perhaps in some situations more powerful than other interventions that can be offered to patients. In an era in which many physicians suffer from professional burnout, mindful practice may be the way in which physicians not only heal themselves, but heal their patients as well.

## Figures and Tables

**Table 1:** Studies on the influence of Mindfulness and Compassion-based interventions for improving physician-patient relationships.

Source	Study Type	Outcome
[7,24]	Original Research	Improved physician-patient communication
[42]	Intervention Program	Improved patient care and satisfaction based on patients reports Enhanced psychosocial orientation
[63]	Original research	Improved empathy and quality of care based on patients reports
[64]	Review	Enhanced empathy
[65]	Original research	Increased self-regulation and self-compassion
[66]	Original research	Increase in patients’ satisfaction and care
[67]	Conceptual model	Improved patient-centered communication
[49]	Original Research	Increased patient-centered care Patients reported being better understood
[13]	Original Research	Increased helping behavior
[17]	Original Research	Enhanced quality of care based on patients reports
[68]	Intervention Program (Focus Group)	Improved empathy, listening skills and quality of care
[23]	Systematic Review	Increased empathy among medical students, residents and physicians
[46]	Original Research	Reduced physicians’ burnout and Increased empathy
[69]	Review	Increased empathy
[47]	Original Research	Increased empathy
[70]	Original Research	Increased empathy and compassion
[71]	Original Research	Increased compassion, empathy and altruism
[72]	Clinical Case	Increased patient understanding
[73]	Original Research	Increased patients’ confidence
[74]	Systematic Review	Empathic accuracy and prosocial behavior

**Table 2:** Mediating mechanisms for the efficacy of MBIs.

Mediating Mechanisms
Relaxation Reappraisal/Reperceiving Metacognition Acceptance Self-compassion Positive emotional states (e.g., “savoring”, “flourishing”) Emotional self-regulation Finding new meanings (values) and sense Developing new resources (e.g., resiliency) Self-transcendence Brain changes (neurofunctional and structural)

Note: Source: [69,70,83,84,85,86,87,88].

**Table 3:** Steps to manage difficulties in physician-patient communication.

Steps for Improving Physician-Patient Communication
Recognizing the existence of one or more barriers, implicit or explicit, in the interaction with the patient. Defining the barriers concretely through different hypotheses-testing and data exploration. Sharing this process with the patient, making him/her participant of the origins of the barrier. Taking action to cope with the barrier or obstacle: working as a team with the patient to establish priorities and make decisions, using active listening and empathy.

Note: Source: [98,99,100].

**Table 4:** Three-minute breathing space.

Three-Minute Breathing Space
Step 1: Becoming Aware
Begin by deliberately adopting an erect and dignified posture.
Close your eyes. Then, bringing your awareness to your inner experience, ask: What is my experience right now?
− What thoughts are going through the mind? Notice them as passing mental events. − What feelings are here? Turn your attention in particular toward any emotional discomfort or unpleasant feelings. − What body sensations are here right now? Scan the body quickly to notice any tightness or bracing.
[PAUSE]
Step 2: Gathering
Bring your attention to the breath. Notice the belly rising on an in-breath and falling on the out-breath. Try to follow it closely for a few cycles to further bring your attention into the present.
[PAUSE]
Step 3: Expanding
Now expand your awareness beyond your breath to take in a sense of your body as a whole, including your posture and facial expression. Notice and breathe into any areas of tension, and let go of the tension with each out-breath. Allow your body to soften and open. You might suggest to yourself, “It’s okay… whatever it is, it’s already here: let me feel it”.
As best you can, bring this accepting awareness into the next moments of the day.
Adapted from Williams et al. [101].
